# Investigating the association between work family conflict (WFC) and generalized anxiety disorder (GAD) in an Australian community-based cohort study

**DOI:** 10.1007/s00127-024-02672-8

**Published:** 2024-05-23

**Authors:** Tianying Wang, Peter Butterworth, Amanda Cooklin, Lyndall Strazdins, Liana Leach

**Affiliations:** 1 National Centre for Epidemiology and Population Health (NCEPH), The Australian National University, Canberra, 2601 Australia; 2 Judith Lumley Centre, La Trobe University, Melbourne, Australia

**Keywords:** Work family conflict (WFC), Anxiety, Psychological job characteristics

## Abstract

**Purpose:**

Difficulties managing work and family demands are common and have been found to be associated with stress and poor mental health. However, very few studies have examined Work Family Conflict (WFC) in connection with diagnosable anxiety disorders (and none with Australian representative data). The current study investigated whether high WFC was significantly associated with a diagnosis of Generalised Anxiety Disorder (GAD) after controlling for a broad range of socio-demographic contextual factors, related psychosocial job, family and individual characteristics, and prior anxiety symptom history.

**Methods:**

Data was analysed from an Australian population-based community cohort - the Personality and Total Health (PATH) Through Life project. Eligible participants (*N* = 1159) were employed full-time or part-time, with data collected by both online questionnaire and face-to-face interview. Presence of Generalised Anxiety Disorder (GAD) in the past 12-months was diagnosed by the GAD module in the Composite International Diagnostic Interview (CIDI) (based on DSM-IV criteria) and severe anxiety symptoms were measured using the Patient Health Questionnaire (PHQ) 7-item ‘other anxiety’ model.

**Results:**

The findings consistently showed that those experiencing high WFC had higher odds of a GAD diagnosis (final adjusted model: CIDI: OR: 2.55, CI: 1.38–4.70) as well as clinical levels of anxiety symptoms (PHQ: OR:2.61, CI:1.44,4.72). This was the case after controlling for an extensive range of covariates.

**Conclusions:**

This is one of the first studies to show that WFC is associated with greater likelihood of GAD. The challenge of juggling both work and family can have far-reaching impacts - not just increasing distress broadly, but also potentially increasing the likelihood of clinically diagnosable anxiety.

## Introduction

In recent decades, employment patterns have changed. Some of these changes include working longer hours, greater intensity (but also flexibility) in work schedules, and a growing proportion of women participating in the labour market [1]. In Australia, about 20% of non-employed women transition into work every year, and the proportion of dual-earner couples (where both parents are employed) with at least one child under 18 years of age has dramatically increased over the last 50 years (reaching 68% of all couple families with children in 2018) [2]. This increase in dual work and family role obligations is matched by a growing body of research demonstrating the prevalence and adverse consequences of Work Family Conflict (WFC).

WFC describes the pressure and dissonance that comes from trying to manage conflicting or incompatible work and family demands [1, 3]. WFC is a widespread hazard, with one study finding that around 50% of Australian parents report often needing to abandon important family activities due to work [4]. In the work-family literature, WFC is viewed as a chronic role strain or stressor that could be reduced if individuals were better supported to balance multiple roles in their work and family [5]. WFC is both related-to, but also independent of, other job adversities such as high job demands, job insecurity and lack of job flexibility [6].

### WFC and mental health

Early research in the work-family literature consistently identified correlations between WFC and broad, self-reported, dimensional measures of psychological strain [7]. Psychological strain has been proposed to include three main elements: distress, depression and anxiety [8]. The scales adopted in these (usually small) studies provide a dimensional (i.e. continuous) assessment of nonspecific symptoms of stress, anxiety and depression, and foregrounded later work looking specifically at WFC and depression and/or anxiety symptomology. More recent, large, population-based studies consistently show a positive association between WFC and distress. For example, cross-sectional research in the US using nationally representative data from the National Health Interview Survey (NHIS) showed that WFC was independently associated with increased odds of psychological distress (assessed using the K6 psychological distress scale) after adjusting for a range of occupational, behavioural and socio-demographic influences [9].

#### WFC and Depression

While not as common as research using a general strain or distress measure, other research has focused on the connection between WFC and levels/symptoms of depression. Studies analysing data from large, representative samples have shown that higher WFC is associated with greater depression symptoms [10, 11]. A small number of studies have investigated the association between WFC and a diagnosis of major mood or depressive disorder (MDD), finding that higher WFC is associated with greater likelihood of depressive disorder [12–15]. While WFC has been shown to be associated with depression and general distress, little research has addressed a possible link with anxiety. Given there are strong indications in the literature that WFC is connected to elements of anxiety such as somatic and psychosomatic complaints, and time pressure and strain [7, 16]– [19], there is a need to further understand the extent of this relationship.

#### WFC and anxiety:

Existing research examining WFC and anxiety has largely been limited to certain vocations or populations, with studies using continuous rather than diagnostic measures. For example, a study by Zhang et al. (2020) analysed data from 764 female health care workers in China [20], and research by Yu and Zi (2020) analysed data from 986 coal miners in China [21], both finding WFC is associated with more self-reported anxiety symptoms. While there are a small number of studies suggesting there is a relationship between WFC and anxiety, further research showing that WFC is linked to anxiety disorders specifically would help make the case that incompatible work and family pressures are an important, independent, social determinant of anxiety. Research focused on anxiety disorders could help respond to queries regarding the high correlation/overlap between WFC and broad continuous anxiety (and distress) measures, as would research that controls for other influential work and family-related correlates.

### Advancing research on WFC and anxiety disorder diagnosis

Diagnosis of anxiety disorders are related to, but different from, symptom levels or counts of anxiety (and psychological distress), as they reflect a longer duration and greater severity of symptoms. Generalised Anxiety Disorder (GAD) in particular is characterized by excessive worry over a long period of time [22]. According to the most recent version of the Diagnostic and Statistical Manual of Mental Disorders (DSM-V), the primary criteria is that anxiety and worry occur over at least six months and are associated with other relevant symptoms (e.g., fatigue and irritability) [23]. The anxiety, worry, and/or physical symptoms must result in clinically severe distress or impairment in essential dimensions of life such as social and occupational domains, and these disturbances cannot be explained by any other medical conditions [23].

There is evidence from a handful of representative national studies that WFC is associated with higher odds of an anxiety disorder diagnosis. For example, Grzywacz & Bass (2003) analysed cross-sectional data from the National Survey of Midlife Development in the US (MIDS) and found that WFC was associated with greater odds of an(y) anxiety disorder assessed using the Composite International Diagnostic Interview Short Form Scales (CIDI-SF) [24], however this assessment was a brief self-report measure rather than a complete diagnostic assessment. Frone (2000), used data from the US National Comorbidity study (*n* = 8,098) to evaluate the relationship between WFC and specific types of psychiatric disorders, as measured by the in-person clinical Composite International Diagnostic Interview (CIDI) [14]. The study found that individuals who are exposed to WFC are more likely to experience an(y) anxiety disorder [14]. It is important to note that while these large national studies controlled for broad socio-demographic factors such as gender, age, marital status, education and income, they did not control for other psychosocial factors in the workplace that are known to be associated with psychological distress, depression and anxiety, nor for other potentially related individual characteristic (such as personality) [14, 20, 21, 24, 25].

A cross-sectional study by Murcia, Chastang, and Niedhammer (2013) is one of the only studies to have specifically investigated work-life imbalance related to GAD. This study initially found that there was a higher risk, especially for women (based on a national sample of French workers (*n* = 13,669)). In this study, GAD was measured by the Mini International Neuropsychiatric Interview, which was developed from the DSM-IV criteria [26], however WFC was only measured by a single-item. The findings showed that once a range of workplace characteristics, including psychosocial adversities such as job strain, social support, job security, were controlled there was no longer a significant association between WFC and GAD. Further research is needed to build upon and extend Murcia et al.’s findings – including using a validated measure of WFC and adjusting for prior anxiety history. In addition, gender differences in the impacts of WFC on anxiety disorder diagnosis have not thoroughly been explored in the small number of population-based studies conducted (e.g. Grzywacz & Bass 2004; Frone 2000; Wang 2006) nor has the potential impacts of parenting responsibilities.

### Aims of the current study

Very few studies that have used representative community-based data to examine the association between WFC and diagnosable anxiety disorders. While these indicate that greater WFC is associated with increased likelihood of a disorder, the only study which has controlled for other influential psychosocial job characteristics (i.e. Murcia et al. 2013) found that any association is explained by these other workplace factors [26]. It appears no Australian research has investigated WFC in connection with diagnosis of anxiety disorders.

Thus, Aim 1 of this study was to use a large representative Australian community sample to examine the relationship between WFC and GAD (as diagnosed using the CIDI diagnostic interview) after adjusting for various social-demographic factors, psychosocial job characteristics, personality. We control for previous symptoms levels of anxiety (at an earlier wave of data collection) to strengthen our investigation of WFC specifically as a potential risk factor for GAD. Aim 2 was to examine the association between WFC and anxiety symptom levels using a well-validated anxiety scale to test the consistency of findings. Aim 3 investigated potential gender differences and the influence of children in the association between WFC and GAD (as these may both be potential moderating factors).

## Method

### Participants

This study analyses data from the most recent Wave of the Personality and Total Health (PATH) Through Life project data (Wave 5). PATH is unique as a longitudinal, population-based community cohort dataset [27], which at Wave 5 included measures of WFC and other psychosocial job characteristics as well as diagnostic information for GAD. Initially at Wave 1, participants were recruited (at random) from the electoral records of Canberra, ACT, and Queanbeyan, NSW, Australia, with participants eligible if initially in one of three age groups: 20s (20–24 years), 40s (40–44 years), and 60s (60–64 years). Follow-up data has been gathered from these participants at around four-yearly intervals, with five waves of data obtained thus far [28].

The current study focuses on the 20s cohort (aged 20–24 at Wave 1 and 38–43 at Wave 5). At Wave 1 (in 1999), the baseline survey was completed by 2404 respondents. The current study is based on 1257 participants who remained in the study at Wave 5 (in 2017), and who completed both an online survey and the additional face-to-face interview. The final group included the sample for analysis were 1159 respondents who reported that they were full-time or part-time employed. The missing data on any variable included in the analyses was less than 2%, and thus no data imputation was applied.

### Measures

#### Work family conflict

The Wave 5 survey included four questions that captured two aspects of WFC – work-to-family conflict and family-to-work conflict: (1) *Because of my work responsibilities: (a) I have missed out on home or family activities that I would like to take part in, and (b) My family time is less enjoyable and more pressured. (2) Because of my family (including caring) responsibilities: (a) My work time is less enjoyable and more pressured and (b) I have to turn down work activities or opportunities that I would prefer to take on*. These questions were based on Marshall and Barnett’s (1993) measure of strains between work and family. These items, or similar, have been asked in numerous studies investigating WFC and anxiety (e.g. Frone 2000) [14, 29], and in a nationally representative Australian government-funded cohort study [30]). Response to each question ranges from 0 (strongly disagree) to 4 (strongly agree). All responses were summed, with a total scale ranged from 0 to 16. A cut-off point of > 7 (i.e. at the upper tertile) was used to classify high levels (1) of WFC versus lower levels of WFC (0). The Cronbach’s alpha of the 4 items was 0.7195.

#### Anxiety measures

##### Anxiety symptoms:

Anxiety symptoms were measured using a short screening measure that assesses anxiety severity within the Patient Health Questionnaire (PHQ) (i.e. the self-administered version of the Primary Care Evaluation of Mental Disorders (PRIME-MD)) [31], . The short screening tool includes 7-items designed to assess “other anxiety” (separate from panic disorder, which is another module within the PHQ). This “other anxiety” scale shares significant similarities in items with the more specific GAD-7 scale (developed subsequently to focus specifically on GAD) with some additional focus on somatic symptoms. The scale has been shown to relate to GAD and anxiety disorders more generally [32], . All participants are asked an initial question (“*how often have you been bothered by feeling nervous, anxious, on edge, or worrying a lot about different things in the past four weeks*”), and those who answer on “several days” or “more than half the days” are subsequently asked six anxiety-related questions with the answers “yes” (score = 1) and “no” (score = 0). Once a total scale score was generated, a cut-off point (score > = 3) was used to indicate a clinical level of anxiety (representing a likely anxiety disorder) (coded 1) vs lower anxiety (coded 0) [33].

##### GAD Diagnostic interview:

During the face-to-face interview, interviewers implemented the GAD module of the Composite International Diagnostic Interview (CIDI), with participant responses used to generate a diagnosis of 12-month Generalised Anxiety Disorder (GAD). The CIDI items were coded according to the Diagnostic and Statistical Manual of Mental Disorders-IV [22, 34]. More specific than the broader PHQ ‘other anxiety’ screening scale, the DSM-IV classifies diagnosis of GAD when people have experienced excessive anxiety and worry with more than two symptoms for at least six months.

#### Covariates

A range of sociodemographic variables known to be associated with WFC and anxiety were considered. These variables included gender, number of negative life events, education, income, presence of children (< 5 years), work hours, relationship status, housework distribution, physical health problems, smoking and alcohol use, personality, and anxiety symptoms in a previous wave of data collection (Wave 3) [30, 35].

*The number of negative life events* were summed and divided into three categories: none (coded 0), one (coded 1), and two or more events (coded 2+). Participants were asked about negative life events during the past 6 months using an extended version of the List of Threatening Experiences Questionnaire [36] and questions from the British National Survey of Health and Development [37]. Analyses included 16 items about adverse events in the past six months, including serious illness, injury, assault of a close relative or yourself, death of a close family member or friend, relationship separation, serious problems within close relationships, financial crisis, work disappointments, legal problems, marital difficulties, and loss of something valuable.

*Housework distribution* was assessed by asking whether the participant was 100%, 75%, 50%, 25% or 0% responsible for housework. These responses were coded into three categories: 100% responsibility (indicating higher role strain in domestic duties) (coded 1), 50-75% responsibility (coded 2), and less than 50% responsibility (coded 3).

*Work hours* were recoded to represent short part-time hours (less than 30 h) (coded 1), medium part-time to full-time hours (30–49 h) (coded 2) and long-full time hours (over 50 h or equivalent) (coded 3).

*Income levels* were coded into three categories: low-income group (less than $1075 per week) (coded 1), median-income group ($1075-$3800/week) (coded 2), and high-income group (more than $3800 per week) (coded 3).

*Smoking status and alcohol behaviour* was asked about using the following items: *“do you currently smoke?”* and *“how do you classify your drinking behaviour?”* with the possible responses to the alcohol item “abstain” (0), “occasional” (1), “light” (2), “medium” (3), “hazardous” (4), and harmful (5). The response to the smoking item were grouped into “current smokers” (coded 1) and “not current smokers” (coded 0). The responses to the alcohol item were grouped into “abstaining or moderate drinkers” (scores 0 to 3 coded as 0) and “hazard drinkers” (scores 4 to 5 coded as 1).

*Physical health problems* were derived from self-reported checklist of chronic physical health disorders included epilepsy, asthma, chronic bronchitis, emphysema, diabetes, thyroid, heart problems, arthritis, and Parkinson’s Disease. This variable was coded into binary categories with respondents with no medical conditions labelled as “no conditions” (coded 0), and those with any medical condition coded as “at least 1 condition” (coded 1).

*Psychosocial job characteristics* were evaluated in four areas: job demands, job decision/control discretion, job skill discretion and job security [38]. There were four items assessing *job demands* such as “*Do you have to work very fast?*” *Job decision* or job decision/control was assessed by nine items such as “*Do you have a choice in deciding how you do your job?*”. Job skill represented job skill discretion using six items such as “*Does your job provide you with a variety of interesting things?*” All the above questions included four response options: “often”(4), “sometimes”(3), “rarely”(2) and “never”(1), and responses were summed within each psychosocial job domain to form a scale where higher scores represented lower job quality. A tertile cut-off point was then used to determine “high” (coded 1) and “low” (coded 0) job demands, job control and job skill. *Job security* was evaluated by a single item – “*How secure do you feel about your job or career future in your current workplace?*” with four response categories scored from 1 to 4 (“not at all secure”, “moderately secure”, “secure”, “extremely secure”). Respondents with score 3 and 4 were classified as having high (coded 1) job security, with responses 1 and 2 classified as low (coded 0) job security [39].

*Personality (emotional stability)* was measured by the Ten-Item Personality Inventory (TIPI) [40]. There were 10 items from the TIPI included in the questionnaire, with two specifically assessing emotional stability. The items are “I see myself as” (a) “anxious and easily upset”; (b) “calm and emotionally stable”. These two questions were responded to on a 7-point scale ranging from 1 “Disagree strongly” to 7 “Agree strongly”. These items were summed to provide a total score ranging from 2 to 14 where a higher score represented less emotional stability.

*Anxiety symptoms in Wave 3* were measured by seven items in the PHQ ‘other anxiety’ module (as explained above) using the same cut-points as previously described (i.e. the same items used in Wave 5).

### Statistical analyses

Descriptive information was calculated for the overall sample, and chi-square tests examined the relationship between the socio-demographic covariates and WFC. A series of logistic regression analyses were conducted to examine the association between WFC and anxiety for the two different anxiety assessments: DSM-IV criteria for GAD based on the CIDI diagnostic interview (Aim 1) and symptom levels based on the PHQ anxiety measure (Aim 2). For each anxiety measure, four models were run sequentially. The first was an unadjusted Model 1. Model 2 adjusted for psychosocial job quality characteristics. The socio-demographic covariates were included in Model 3. The final model adjusted for history of anxiety in Wave 3 (to account for anxiety prior to the onset of WFC). Interaction tests explored whether the relationship between WFC and anxiety might be moderated by parent gender or the presence of children. All analyses were run with all available data in each model, using STATA^MP^ Version 17.0.

## Results

Characteristics of the sample and the univariate associations between sociodemographic factors, other covariates, the anxiety binary categories and WFC (low vs. high) are presented in Table 1. All participants were employed (*N* = 1159), and were aged between 37 and 43 years, with 54% female and 86.6% with a bachelor’s university degree or higher. Most (79%) of the sample were married or living with a partner, and while 21% of the sample did not have children, 34% had at least one child under five years old. The majority (62%) worked 30 to 49 h per week. 59% of participants were affected by at least one negative life event in the past 6 months. Over 32% of participants reported at least one physical health problem such as diabetes or asthma, and 9% consumed alcohol at hazardous levels. For the PHQ measure, 7.1% of people scoring over the cut-point indicating a clinical level of anxiety. Using the DSM-IV diagnostic criteria as measured by the CIDI, 6% of participants met the criteria for GAD.

**Table 1:** Socio-demographic characteristics of respondents all at wave 5 (*N* = 1159)

Sample characteristic	All participants	Low WFC (*n* = 727)	High WFC (*n* = 428)	Chi-square test (Low vs. High WFC)
	n (%)	n (%)	n (%)	*p*-value
Gender				0.279
Male	530 (45.7)	343 (47.2)	184 (43.0)	
Female	628 (54.2)	383 (52.7)	244 (57.0)	
Other	1 (0.1)	1 (0.1)	0	
Missing	0			
Children				< 0.001
No children	246 (21.2)	183 (25.3)	61 (14.4)	
Have children < 5Yrs	405 (34.9)	220 (30.4)	184 (43.3)	
Have children > 5Yrs	501 (43.2)	321 (44.3)	180 (42.3)	
Missing	7 (0.6)			
Work hours				0.749
Short Part-time (< 30 h)	187 (16.1)	119 (16.5)	68 (16.0)	
Medium Part-time to Full time (30–49 h)	718 (62.0)	454 (62.9)	262 (61.5)	
Long Full time ( > = 50 h)	245 (21.1)	149 (20.6)	96 (22.5)	
Missing	9 (0.8)			
Life events				0.023
0	465 (40.1)	306 (42.3)	158 (37.1)	
1	303 (26.1)	198 (27.3)	105 (24.6)	
2+	384 (33.1)	220 (30.4)	163 (38.3)	
Missing	7 (0.6)			
Income				0.427
High (>$3800/w)	285 (24.6)	172 (24.6)	113 (27.6)	
Medium ($1075-$3800/w)	729 (62.9)	462 (66.2)	266 (64.9)	
Low (<$1075/w)	96 (8.3)	64 (9.2)	31 (7.6)	
Missing	49 (4.2)			
Married status				0.003
Yes, living with someone	917 (79.1)	556 (76.6)	358 (84.0)	
No, or living separate	239 (20.6)	170 (23.4)	68 (16.0)	
Missing	3 (0.3)			
Education level				< 0.001
Completed or below Yr12	152 (13.1)	106 (14.6)	46 (10.8)	
Below bachelor’s degree	329 (28.4)	234 (32.3)	93 (21.8)	
Bachelor’s degree or higher	675 (58.2)	385 (53.1)	288 (67.4)	
Missing	3 (0.3)			
Housework distribution				0.185
100% responsible	224 (19.3)	151 (20.8)	72 (16.8)	
>=50% responsible	789 (68.1)	492 (67.7)	297 (69.4)	
< 50% responsible	144 (12.4)	84 (11.5)	59 (13.8)	
Missing	2 (0.2)			
Physical Health Problems				0.133
N o	770 (66.4)	495 (68.6)	273 (64.2)	
Yes	379 (32.7)	227 (31.4)	152 (35.8)	
Missing	10 (0.9)			
Personality-Emotional stability^a^		Mean (SE)	Mean (SE)	< 0.001
Score 2–14	1151 (99.7)	10.20 (0.10)	9.57 (0.14)	
Missing	8 (0.7)			
Smoking				0.090
No	1029 (88.8)	638 (87.9)	389 (91.1)	
Yes	126 (10.9)	88 (12.1)	38 (8.9)	
Missing	4 (0.3)			
Alcohol				0.798
No	1052 (90.8)	662 (91.3)	388 (90.9)	
Yes (Harmful)	102 (8.8)	63 (8.7)	39 (9.1)	
Missing	5 (0.4)			
Job skills				0.188
Low	710 (61.3)	457 (63.2)	252 (59.3)	
High	440 (38.0)	266 (36.8)	173 (40.7)	
Missing	9 (0.7)			
Job demands				< 0.001
Low	759 (65.5)	533 (73.7)	225 (52.9)	
High	391 (33.7)	190 (26.3)	200 (47.1)	
Missing	9 (0.8)			
Job decision/autonomy				< 0.001
Low	736 (63.5)	435 (60.2)	300 (70.4)	
High	415 (35.8)	288 (39.8)	126 (29.6)	
Missing	8 (0.7)			
Job security				0.002
Low	327 (28.2)	182 (25.2)	143 (33.6)	
High	824 (71.1)	541 (74.8)	283 (66.4)	
Missing	8 (0.7)			
Anxiety-CIDI (12 months)				< 0.001
No	1087 (93.8)	701 (96.4)	382 (89.9)	
Yes	69 (6.0)	26(3.6)	43(10.1)	
Missing	3 (0.2)			
Anxiety- PHQ-MD (Wave 5)^b^				0.001
No	1073 (92.6)	689 (94.9)	382 (89.5)	
Yes	82 (7.1)	37 (5.1)	45 (10.5)	
Missing	4 (0.3)			
Anxiety- PHQ-MD (Wave 3)^b^				0.330
No	1070 (92.3)	674 (94.1)	392 (92.67)	
Yes	73 (6.3)	42 (5.9)	31 (7.33)	
Missing	16 (1.4)			

a. a higher score represented less emotional stability

b. cut-off points (scores > = 3)

c. The prevalence of the psychosocial job quality variables (job skills, demands, autonomy) was created by using the upper tertile a cut-point (see Methods section for further details)

Significant univariate associations with high WFC are presented in Table 1. Participants were significantly more likely to have high WFC if they were married or living with a partner, had children under five years old, had a bachelor’s degree or higher, if they experienced two or more negative life events, and if they scored lower on emotional stability. As for psychosocial job characteristics, those with higher job demands, higher job decision/control ability or lower job security were more likely to report high WFC. Also, scoring above the cut-point indicating clinical levels of anxiety on the PHQ, and meeting the CIDI criteria for GAD, was associated with high WFC.

Logistic regression results for the associations between WFC and the two different anxiety measures are shown in Table 2. In relation to study Aim 1 using CIDI diagnosis, the unadjusted model (Model 1) found that those with high WFC had higher odds of being diagnosed with GAD (OR: 3.03, CI: 1.84–5.02). The effect of WFC in association with GAD was slightly reduced in subsequent models but remained significant in the final model (Model 4) after adjusting for all covariates including other psychosocial job quality factors and prior anxiety (OR: 2.55, CI:1.38–4.70). As for Aim 2, the unadjusted model for the PHQ anxiety outcome also showed that the odds of clinical symptom levels for anxiety were higher when WFC was high (OR: 2.19, CI: 1.40–3.45) and this result remained significant after all adjustments in the final model (Model 4) (OR:2.61, CI:1.44–4.72). The only other covariates to predict clinical anxiety levels across both measurements after adjusting for other factors were emotional stability and prior high anxiety at Wave 3.

**Table 2:** The relationship between anxiety and work-family conflict in adjusted logistic regression models (*N* = 1159)

	PHQ-MD			CIDI- 12 months		
	Model 2^a^ (*N* = 1148)	Model 3^b^ (*N* = 1095)	Model 4^c^ (*N* = 1079)	Model 2^a^ (*N* = 1145)	Model 3^b^ (*N* = 1093)	Model 4^c^ (*N* = 1077)
WFC (High)	1.97**(1.23, 3.16)	2.48**(1.38, 4.36)	2.61**(1.44, 4.72)	2.83***(1.68, 4.76)	2.38**(1.31, 4.35)	2.55**(1.38, 4.70)
Job demands (High)	1.14(0.69, 1.88)	1.55(0.84, 2.83)	1.45 (0.78, 2.71)	0.79 (0.45, 1.38)	1.04 (0.55, 1.99)	1.07 (0.56, 2.05)
Job skill (High)	0.52*(0.29, 0.92)	0.78(0.41, 1.49)	0.73 (0.37, 1.41)	0.79(0.44, 1.42)	1.15 (0.59, 2.21)	1.05 (0.54, 2.05)
Job decision/ autonomy (High)	0.65(0.37, 1.14)	0.98(0.51, 1.88)	1.01 (0.52, 1.96)	0.49*(0.26, 0.94)	0.70 (0.34, 1.44)	0.72 (0.35, 1.48)
Job security (High)	0.37***(0.23, 0.59)	0.67(0.38, 1.18)	0.66 (0.38, 1.17)	0.49**(0.30, 0.82)	0.90 (0.49, 1.63)	0.86 (0.47, 1.57)
Gender (Male)		0.72(0.37, 1.38)	0.73 (0.38, 1.44)		0.68 (0.34, 1.38)	0.67 (0.33, 1.36)
Life events (0)						
1		1.23(0.54, 2.81)	1.28 (0.56, 2.94)		0.88 (0.39, 2.00)	0.84 (0.36, 1.93)
2+		2.44*(1.22, 4.89)	2.55*(1.26, 5.18)		1.65 (0.82, 3.31)	1.67 (0.83, 3.38)
Income (High)						
Medium		0.55(0.25, 1.22)	0.49 (0.22, 1.11)		0.55 (0.23, 1.34)	0.59 (0.24, 1.47)
Low		0.69(0.23, 2.02)	0.58 (0.19, 1.77)		0.27*(0.08, 0.95)	0.30 (0.09, 1.08)
Children (No)						
Children <5Yrs		0.82(0.35, 1.92)	0.79 (0.33, 1.89)		1.03 (0.41, 2.59)	0.98 (0.39, 2.46)
Children > 5Yrs		1.22(0.57, 2.61)	1.24 (0.56, 2.74)		1.49 (0.64, 3.46)	1.33 (0.56, 3.13)
Work hours (Short)						
Medium		0.63(0.32, 1.27)	0.63 (0.31, 1.28)		0.80 (0.39, 1.62)	0.76 (0.37, 1.55)
Long		0.56(0.22, 1.45)	0.59 (0.22, 1.57)		0.57 (0.19, 1.64)	0.59 (0.20, 1.72)
Married status (No)		1.62(0.58, 4.52)	1.40 (0.47, 4.22)		0.82 (0.26, 2.53)	0.67 (0.21, 2.16)
Housework distribution (100%)						
> 50% responsible		1.59(0.60, 4.19)	1.55 (0.55, 4.31)		0.71 (0.26, 1.94)	0.59 (0.21, 1.67)
< 50% responsible		2.39 (0.68, 8.37)	2.25 (0.62, 8.21)		0.25 (0.04, 1.49)	0.21 (0.04, 1.30)
Education level (< Yr12)						
Bachelor		1.28 (0.60, 2.75)	1.30 (0.60, 2.84)		0.55 (0.23, 1.35)	0.57 (0.23, 1.40)
Over Bachelor		0.50 (0.23, 1.12)	0.54 (0.24, 1.21)		1.03 (0.46, 2.31)	1.01 (0.45, 2.27)
Smoking (Yes)		1.96 (0.95, 3.87)	2.12* (0.98, 4.15)		0.99 (0.41, 2.40)	1.14 (0.42, 2.78)
Alcohol (Yes)		0.61 (0.24, 1.55)	0.66 (0.33, 1.03)		0.38 (0.12, 1.15)	0.28* (0.08, 0.97)
Physical health problems (Yes)		1.80*(1.04, 3.02)	1.77 (0.97, 2.90)		1.26 (0.71, 2.24)	1.22 (0.68, 2.18)
Personality – Emotional stability		0.70***(0.63, 0.78)	0.73***(0.66, 0.81)		0.72***(0.64, 0.80)	0.71***(0.63, 0.79)
Anxiety in wave 3			3.40**(1.71, 6.77)			1.02 (0.56, 1.86)

*Notes: odds ratios and confidence intervals displayed. *p* < .05* *p* < .01** *p* < .001***. Unadjusted model (Model 1 in Results text): PHQ (*N* = 1153), 2.19**(1.40, 3.45); CIDI-12 months (*N* = 1152), 3.03***(1.84, 5.02)

a. Model 2: adjusted for psychosocial job quality variables

b. Model 3: add socio-demographic and contextual covariates

c. Model 4: add the history of anxiety in Wave 3

To address Aim 3, brief interaction analyses explored whether the results might differ based on participants’ gender and whether they had children. First, we note that the chi-square tests in Table 1 show that there were no gender differences in the proportion of the sample with high WFC (*p* = .279), but that those with high WFC were significantly more likely to have children (*p* < .001). Interactions tested between WFC and gender for each of the anxiety outcomes, showed that gender did not moderate the associations. Interactions testing whether children might moderate any association found that the association between WFC and anxiety was stronger for parents than those without children, for both the GAD and PHQ outcomes (Model 4 interaction terms: GAD: OR: 2.43, CI: 1.22–4.83; PHQ-MD: OR: 3.13, CI: 1.59–6.16). When follow-up analyses explored an interaction based on children’s age (i.e. 5 years or under) they showed no statistically significant interactions between WFC and children’s age specifically.

## Discussion

This is the first population-based study conducted in Australia to investigate the association between work-family conflict (WFC) and clinical anxiety as indicated by a DSM-diagnosis of Generalised Anxiety Disorder (GAD). The results show that experiencing high WFC is strongly related to GAD diagnosis (stronger than other well-established risk factors included in the analyses). The analyses controlled for family factors, other job characteristics, personality and importantly a history of anxiety. The study provides new valuable information about how WFC is related to clinically significant mental health disorders (in this case GAD) in the Australian context, with the outcomes confirmed by using multiple measures/criteria for clinical levels of anxiety.

The current study is consistent with previous studies showing that WFC is associated with and harms mental health [30, 41]. However this past research has commonly adopted distress or depression levels as the outcome measures assessed, or has combined measures of anxiety and depression (e.g. Moreira et al. [42]), whereas our study focused on WFC and diagnosis of GAD using clinical interviews. In terms of anxiety specifically, the current findings align with the small number of relevant studies – as nearly all have similarly shown a positive association. The results from other population-based studies using diagnostic criteria for anxiety, also align with the current findings (e.g. Grzywacz & Bass 2004; Frone 2000; Wang 2006 [14, 24, 25]), however the current study goes a step further in the range of covariates controlled for and the inclusion of prior anxiety as a potential confounder (there is very little research with a longitudinal component available). In addition, prior relevant research has only assessed GAD or anxiety symptoms by one criterion or assessment (e.g. Frone 2000 [14]) – rather than providing reliability through multiple assessments as is the case in the current study.

The current study controls for other psychosocial job quality influences to examine the unique contribution of WFC to GAD outside of these other often co-occurring job experiences and characteristics. While our focus is on the findings that WFC is an important contributor above and beyond other influences in the work environment, the results also point towards good psychosocial job quality, particularly job security, as protective against both WFC and anxiety. Other research has similarly shown this to be the case. For example, work looking at the antecedent of WFC has shown poor job conditions, such as high demands and low job flexibility to be important [30]. In the current study, when psychosocial job factors were added into the models together with WFC the impact of WFC was reduced, suggesting a buffering effect that requires further exploration in future research.

In the broader work-family literature, it has often been proposed that women may be more susceptible to both experiencing WFC and adverse psychological effects. Some studies highlight that with greater participation in home and childcare duties, women may be more prone than men to encounter WFC [20, 43]. However, in recent years, several studies also highlight that men are becoming more exposed to the same or even higher levels of WFC than women, and that they also experience its health risks [44, 45]. For example, men’s long working hours generate high levels of WFC, consuming fathers’ energy so that they cannot engage in family activities efficiently [45]. The current study found no gender differences in either the proportion of the sample with high WFC or the strength of the relationship between WFC and anxiety – suggesting WFC and its impacts are globally present and significant for everyone.

The current study did find, similar to prior research, that parents are more likely to report high WFC than people without children. The demands of parenting may generate considerable difficulties for parents in balancing their career and family obligations, and can lead to work-family conflict. Studies have shown that when parents spend more time on providing care for children they experience greater time pressure, boosting WFC [6, 46]. In this study, the association between WFC and GAD was stronger for parents (than those without children), reflecting the considerable impact of the parenting role in connection with work obligations, on clinically relevant and impairing anxiety.

### Limitations and strengths

There are some limitations to this study. First, the sample size of people with GAD was small limiting statistical power. Only 82 and 69 participants met the cut-point or diagnostic criteria for GAD according to the PHQ ‘other anxiety’ scale and CIDI criteria, respectively. However, this reflects the small percentage of people in the general population that meet diagnostic criteria for GAD, and so is consistent with expectations. We also note that the more specific, now commonly used, GAD-7 screener [47], as not available to use in the current study, and that this may have been a more specific/accurate tool than the ‘other anxiety’ module from the PHQ. Our findings of 7.1% and 6.0% for prevalence of GAD using the ‘other anxiety’ and CIDI diagnostic measure are comparable with data from the Australian Bureau of Statistics (ABS). The ABS data suggests a 12-month prevalence rate of GAD of 4% among individuals aged 35 to 44 years [48].

Second, given those with severe anxiety are more likely drop out of employment due to the impacts on every day functioning and routines [49], it is likely that some of the cohort experiencing both high WFC and high anxiety have previously exited the study (i.e. before Wave 5). This process of health selection into the Wave 5 sample may mean the associations reported are underestimated. Third, while this study advances previous literature by adjusting for prior reported anxiety symptoms, a causal association would be best explored using prospective research with multiple data-points assessing both WFC and GAD. Unfortunately, in the current study, only one measure of WFC (at Wave 5) was available, precluding more in depth longitudinal investigations of causality and possible bi-directional or compounding effects. This study also has some important advantages – including a broad population-based sample, adjustment for a broad range of s work and family covariates, and the adjustment for prior anxiety. This study used both clinical diagnostic interviews and anxiety symptom screening methods, which provides more comprehensive findings than previous studies which only use one measurement for GAD or anxiety symptoms identification.

## Conclusion

This study is one of the first to investigate and find that intensive WFC is related to Generalised Anxiety Disorder among Australians – with the results showing a persistent effect even after controlling for a wide range of other co-occurring risk factors at home and in the workplace. The impacts of WFC are potentially far-reaching, as it is clearly associated with distress broadly and as this study suggests, it is also related to an increased likelihood of clinically diagnosable mental health conditions such as GAD. We note that further longitudinal research is needed to tease out the causal directions between WFC and GAD. Reducing WFC should be a priority for workplaces, public health and mental health initiatives.
